# Current News

**Published:** 1897-12

**Authors:** 


### Current News.

### PROGRAMME OF THE ROCHESTER DENTAL SOCIETY, SESSIONS OF 1897-98.

*October* 19, 1897.—Office of Dr. B. S. Hert; essayist, Dr. W. W. Belcher; subject, "The Laboratory." Discussion, headed by Dr. W. W. Smith and Dr. J. H. Beebee. Office incidents, headed by Dr. L. Requa.

*November* 16, 1897.—Office of Dr. R. H. Hofheinz; essayist, Dr. F. J. Woodworth; subject, "Advice to Patients." Discussion, headed by Dr. F. L. Sibley and Dr. L. Requa. Office incidents, headed by Dr. J. S. Furner.

*December* 21, 1897.—Office of Dr. C. T. Howard; essayist, Dr. C. H. Nicholson; subject, "Temperament as Indicated by the Teeth." Discussion, headed by Dr. I. C. Edington and Dr. W. A. White. Office incidents, headed by Dr. B. S. Hert.

*January* 18, 1898.—Office of Dr. C. F. Howell; essayist, Dr. W. H. Barr; subject, "New Remedies and Methods." Discussion, headed by Dr. F. J. Woodworth and Dr. L. H. Gilbert. Office incidents, headed by Dr. F. M. Rood.

*February* 15, 1898.—Office of Dr. J. E. Line; essayist, Dr. R. H. Hofheinz; subject, "Cements and Gutta-Percha." Discussion, headed by Dr. C. T. Howard and Dr. F. H. Lee. Office incidents, headed by Dr. W. A. White.

*March* 15, 1898.—Office of Dr. H. S. Miller; essayist, Dr. J. E. Line; subject, "Pathological Changes in the Dental Pulp." Discussion, headed by Dr. F. French and Dr. B. S. Hert. Office incidents, headed by Dr. W. A. Windell.

*April* 19, 1898.—Office of Dr. C. H. Nicholson; essayist, Dr. J. W. Cowan; subject, "Gold and Alloys." Discussion, headed by Dr. J. H. Beebee and Dr. L. H. Gilbert. Office incidents, headed by Dr. R. Erler.

*May* 17, 1898.—Office of Drs. J. and L. Requa; essayist, Dr. F. A. Greene; subject, "Fracture of the Superior and Inferior Maxilla." Discussion, headed by Dr. H. S. Miller and Dr. J. Requa. Office incidents, headed by Dr. P. H. Smith.

*June* 18, 1898.—Office of Dr. J. W. Cowan; essayist, Dr. C. F. Howell; subject, "The Year's Advancement in Dentistry." Discussion, headed by Dr. C. H. Nicholson and Dr. H. N. Holmes. Office incidents, headed by Dr. F. L. Sibley.

### NORTHERN ILLINOIS DENTAL SOCIETY.

To the President and Members of the National Dental Association, and of the National Association of Dental Examiners.

Gentlemen,—At the Tenth Annual Meeting of the Northern Illinois Dental Society, held at Rockford, Ill., October 20 and 21, 1897, the undersigned were appointed a committee to draft and present to your Associations suitable resolutions with a view to remedy an existing evil regarding the interstate practice of dentistry, and we herewith submit the following for your consideration:

Whereas, A legal practitioner of any one of the United States, who desires to remove to another State, is, under the existing laws, compelled to comply with certain requirements of the dental law of that State; and

Whereas, In many instances such legal practitioner (sometimes of many years' experience) is subjected to a more or less severe theoretical examination, which cannot even be successfully passed by many who are fresh from the college halls; therefore be it

*Resolved*, That the National Association of Dental Examiners and the National Dental Association be, and are hereby requested to enact such rules, or to secure such modification of the dental laws of the various States which, under reasonable restrictions, will enable competent practitioners to remove from one State to another without being compelled to submit to provisions which are eminently unfair to a large number of capable dentists.

Louis Ottofy,

W. N. Taggart,

M. L. Hanaford,

*Committee.*

Attest:
James W. Cormany, *Secretary.*
Dated, November 10, 1897.

The following are the officers of the Northern Illinois Dental Society for 1898:

President, C. B. Helm, Rockford; Vice-President, Louis Ottofy, Chicago; Secretary, James W. Cormany, Mt. Carroll; Treasurer, M. R. Harned, Rockford.

*Executive Committee.*—E. J. Perry, Chicago.

James W. Cormany,
*Secretary.*

